# Connectome-based disentangling of epilepsy networks from insular stereoelectroencephalographic leads

**DOI:** 10.3389/fneur.2024.1460453

**Published:** 2025-01-03

**Authors:** Kathrin Machetanz, Eliane Weinbrenner, Thomas Volkmar Wuttke, Silke Ethofer, Randolph Helfrich, Josua Kegele, Stephan Lauxmann, Michael Alber, Sabine Rona, Marcos Tatagiba, Holger Lerche, Jürgen Honegger, Georgios Naros

**Affiliations:** ^1^Department of Neurosurgery and Neurotechnology, Eberhard Karls University, Tübingen, Germany; ^2^Department of Epileptology, Eberhard Karls University, Tübingen, Germany; ^3^Department of Pediatric Neurology, Eberhard Karls University, Tübingen, Germany

**Keywords:** connectome, functional connectivity, insula, stereoelectroencephalography, epileptic network

## Abstract

**Objective:**

Epilepsy is considered as a network disorder of interacting brain regions. The propagation of local epileptic activity from the seizure onset zone (SOZ) along neuronal networks determines the semiology of seizures. However, in highly interconnected brain regions such as the insula, the association between the SOZ and semiology is blurred necessitating invasive stereoelectroencephalography (SEEG). Normative connectomes on MRI data enable to link different symptoms and lesion locations to a common functional network. The present study applied connectomics to disentangle epilepsy networks from insular SEEG recordings and to describe their relationship to seizure semiology.

**Methods:**

We retrospectively extracted functional networks by normative connectome analysis from 118 insular contacts depicting epileptic discharges during SEEG in 20 epilepsy patients. The resulting epilepsy networks were correlated to the corresponding semiology by voxel-wise regression and multivariate analyses of variances.

**Results:**

Epileptic foci were found in the posterior insula for somatosensory, other sensory and motor seizures, while cognitive and autonomic symptoms were related to the anterior insula. We identified insular connections to the superior temporal gyrus and heschl gyrus in sensory seizures and projections to the somatosensory cortex in somatosensory seizures. Insula-basal ganglia pathways were found in cognitive seizure manifestations, while insular connectivity to fronto-basal regions were strongest in patients with autonomic seizures.

**Conclusion:**

The semiology of seizures is mirrored in the functional connectivity of insular epileptic discharges. Combining SEEG and connectomics could provide additional information about seizure propagation within the epilepsy network and might enable new treatment options in the future like deep brain stimulation.

## Introduction

The propagation of local epileptic activity from the seizure onset zone (SOZ) along functional neuronal networks explains the semiology of seizures (1). Neurological symptoms and signs appear when ictal discharges activate an eloquent cortical area (i.e., symptomatogenic zone, SZ). If the SOZ is in close proximity to a well-defined SZ (e.g., the primary sensory area), then the semiology (e.g., somatosensory aura) has a good lateralizing and localizing value. In contrast, seizures from different seizure onset zones may evolve to the same SZ producing similar clinical symptoms. Additionally, seizures from one SOZ may propagate to different SZ reducing the specificity of the semiology in these cases (2). Consequently, trying to link semiology to a single SOZ may fail.

Nowadays, many neuropsychiatric diseases or movement disorders are considered “network diseases” of interacting brain regions as it became apparent that similar symptoms can result from lesions in different locations (1, 3, 4). For example, ischemic lesions both in the brainstem or the motor cortex lead to similar symptoms (i.e., hemiparesis) because both affect the corticospinal motor network (3). Accordingly, the semiology of seizures is determined by the functional network affected by the SOZ. The implementation of normative connectomes (i.e., the derivation of standardized brain maps from a group of patients/persons) to evaluate diffusion-weighted-imaging (DWI)-based structural and fMRI-based functional connectivity has significantly advanced research in the field of network diseases. This approach enables to link different symptoms and lesion locations to a common functional network (3, 5–7).

In contrast to other forms of focal seizures, seizures with insular SOZ are characterized by an exceptionally heterogenous phenotype (8, 9). The insular lobe is highly interconnected to a variety of cortical and subcortical areas and involved in sensorimotor, autonomic, cognitive, and socio-emotional functions (10, 11). The propagation of epileptic activity along these diverse functional systems explains the heterogeneity in semiology mimicking other focal seizures (8, 9, 12). For instance, high interconnectivity between the temporal lobe and the insula has been attributed to the occurrence of sensoric phenomena (e.g., olfactory, gustatory) in both temporal lobe epilepsy (TLE) and insular epilepsy (9). The blurred association between semiology and SOZ as well as the anatomic location of the insula within the sylvian fissure often lead to misclassification of insular epilepsy by non-invasive means (e.g., scalp electroencephalography, EEG) (10, 13). Instead, invasive recordings with stereoelectroencephalography (SEEG) to the insula are needed for a reliable differentiation between insular and non-insular SOZs (14–16).

We hypothesize that the heterogenous semiology of insular epilepsy is mirrored by a distinct connectivity pattern of the insular SOZ and cortical SZ. The aim of the study was to evaluate cortico-insular networks in insular epilepsy and their relationship to seizure semiology. For this purpose, we overlaid insular epileptic discharges derived from invasive SEEG recordings with functional and structural MR-based connectomes. The resulting epilepsy networks were subsequently related to the actual seizure semiology. This study is the first using this type of analysis to describe the association between cortico-insular networks and seizure semiology.

## Materials and methods

### Clinical data

In a total of 29 patients SEEG electrodes were inserted into the insula at the University of Tuebingen between May 2016 and November 2021. Nine patients were excluded from the analysis because they did not show any ictal epileptogenic discharges of the insular electrode contacts during monitoring. Therefore, this retrospective study enrolled 20 patients (20.3 ± 13.5 years [2.8–42.5], 14 female) who underwent implantation of electrodes for SEEG. SEEG was indicated by the Interdisciplinary Epilepsy Board of our university (i.e., neuroradiologists, neurologists, neurosurgeons) when non-invasive presurgical evaluation with high-resolution MR imaging (MRI), long-term scalp Video-EEG monitoring (VEEG), neuropsychological assessments and detailed patient semiology were insufficient to delineate the SOZ. In total, 7/20 patients (18.6 ± 15.9 years [2.8–42.5], 6 female) were operated using a frame-based (Radionics^®^ Brown-Roberts-Wells, BRW) and 13 patients (21.3 ± 12.6 years [5.9–40.3], 8 female) in a robot-assisted (ROSA One^®^, Zimmer Biomet, Warsaw, USA) procedure.

The exact description of seizure semiology is based on descriptions by patients and their relatives, and the videos recorded during VEEG and SEEG. The classification distinguished seizures with motor (i.e., myoclonic) and non-motor (i.e., somatosensory, other sensory, cognitive, autonomic) focal as well as generalized tonic-clonic seizures according to the recent terminology of seizures and epilepsy (17). While *somatosensory* seizures affected sensations of e.g. the extremities, other *sensory* seizures involved auditory, visual, olfactory or gustatory perceptions. The study was approved by the local ethics committee of the Eberhard Karls University Tuebingen and performed in accordance with the Declaration of Helsinki. Patients' characteristics are summarized in Table 1.

**Table 1:** Patients' details.

**Age**		20.3 ± 13.5 [2.78–42.5]
**Gender (f:m)**		14:6
**Age at seizure onset (years)**		7.8 ± 7.7 [0–25]
**Semiology**		
**Type of seizure**		
*FIAS*		40% (8/20)
*FBTCS*		60% (12/20)
**Symptoms/signs**		
**Motor**	*Hypermotor*	10% (2/20)
	*Myoclonic (MYOCL)*	25% (5/20)
	*Tonic-clonic (TON-CL)*	60% (12/20)
**Non-motor**	*Sensory (SENSO)*	25% (5/20)
	*Somatosensory (SOMASE)*	40% (8/20)
	*Cognitive (COG)*	15% (3/20)
	*Emotional*	10% (2/20)
	*Autonomic (AUT)*	15% (3/20)
**SEEG—SOZ**		
Insular		40% (8/20)
Temporal		25% (5/20)
Frontal		20% (4/20)
Parietal		5% (1/20)
Occipital		0% (0/20)
Multifocal		10% (2/20)
**Outcome in insular SOZ (2nd-stage surgery)**		
ILAE I		83.3% (5/6)
ILAE II		0% (0/6)
ILAE III		0% (0/6)
ILAE IV		0% (0/6)
ILAE V		16.6% (1/6)
ILAE VI		0% (0/6)

FBTCS, focal to bilateral tonic-clonic seizures; FIAS, focal with impaired awareness seizures; ILAE, International League Against Epilepsy; SEEG, stereoelectroencephalography; SOZ, seizure onset zone.

### Neuroimaging, stereotactic planning and surgical procedure

Preoperatively, all patients underwent magnetic resonance imaging (MRI; Siemens Healthineers, Erlangen, Germany) including a high resolution T1-weighted contrast-enhanced MPRAGE sequence (isovoxel 1 mm). Trajectories were planned based on the preoperative MRI using the planning software of the ROSA^®^ robot (ROSA One^®^, Zimmer Biomet, Warsaw, USA; robot group) or the Brainlab iPlan cranial 3.0 software (Brainlab AG, Feldkirchen, Germany; frame group) following general stereotactic principles. An anterior oblique approach (AOA) or posterior oblique approach (POA) was used for the placement of the insular electrodes as described in detail in previous studies (18, 19).

After placing bone fiducials (WayPoint™, FHC, Bowdoin, USA; robot group) or the stereotactic frame (Radionics^®^ Brown-Roberts-Wells, BRW; frame group) on the day of surgery, an additional preoperative 0.5–1.0 mm contrast-enhanced computer tomography (CT) imaging (Siemens Healthineers, Erlangen, Germany) was performed in all patients and fused to the preoperative MRI. The implantation of SEEG electrodes (Dixi Médical, Besançon, France) was performed in an identical standardized manner as described in detail elsewhere (16, 18). In summary, after indicating the entry point with the stereotactic device and performing a drill hole using a motorized 2.1-mm twist drill (Acculan 4, Aesculap AG, Tuttlingen, Germany), the dura was coagulated and an anchor bolt for the electrode was placed. Subsequently, a stylet was introduced for electrode guidance and the electrode was launched and fixed with the adjacent screw to the anchor bolt. Finally, after the implantation of ~8.6 ± 2.7 [4–15] electrodes per patient, bone fiducials (robot group) or the stereotactic frame (frame group) were removed.

Postoperatively, every patient underwent a high-resolution T1-weighted MR scan controlling for final electrode localization and early detection of postoperative complications. An additional high-resolution CT scan was performed, if the MR scan was not available immediately after surgery.

### Epileptiform activity on SEEG

SEEG monitoring was performed in our video-EEG monitoring unit using the Xltek^®^ Brain Monitor amplifier (Natus Medical Incorporated, San Carlos, California, USA) and a 128-channel breakout box. Data was recorded unfiltered with a sampling rate of 512 Hz and filtered for further analyses later. For this purpose, only a high pass filter of 1 or 3 Hz was used. Electroencephalographic activity was analyzed by two experienced epileptologists. Insular contacts that exhibited spikes, sharp waves, spike-and-slow wave complexes, rhythmic activity or low voltage fast activity (LVFA) during observed seizures (ictal) were classified as epileptiform for further analysis.

### Visualization of insular epileptogenic activity

**Figure 1:**
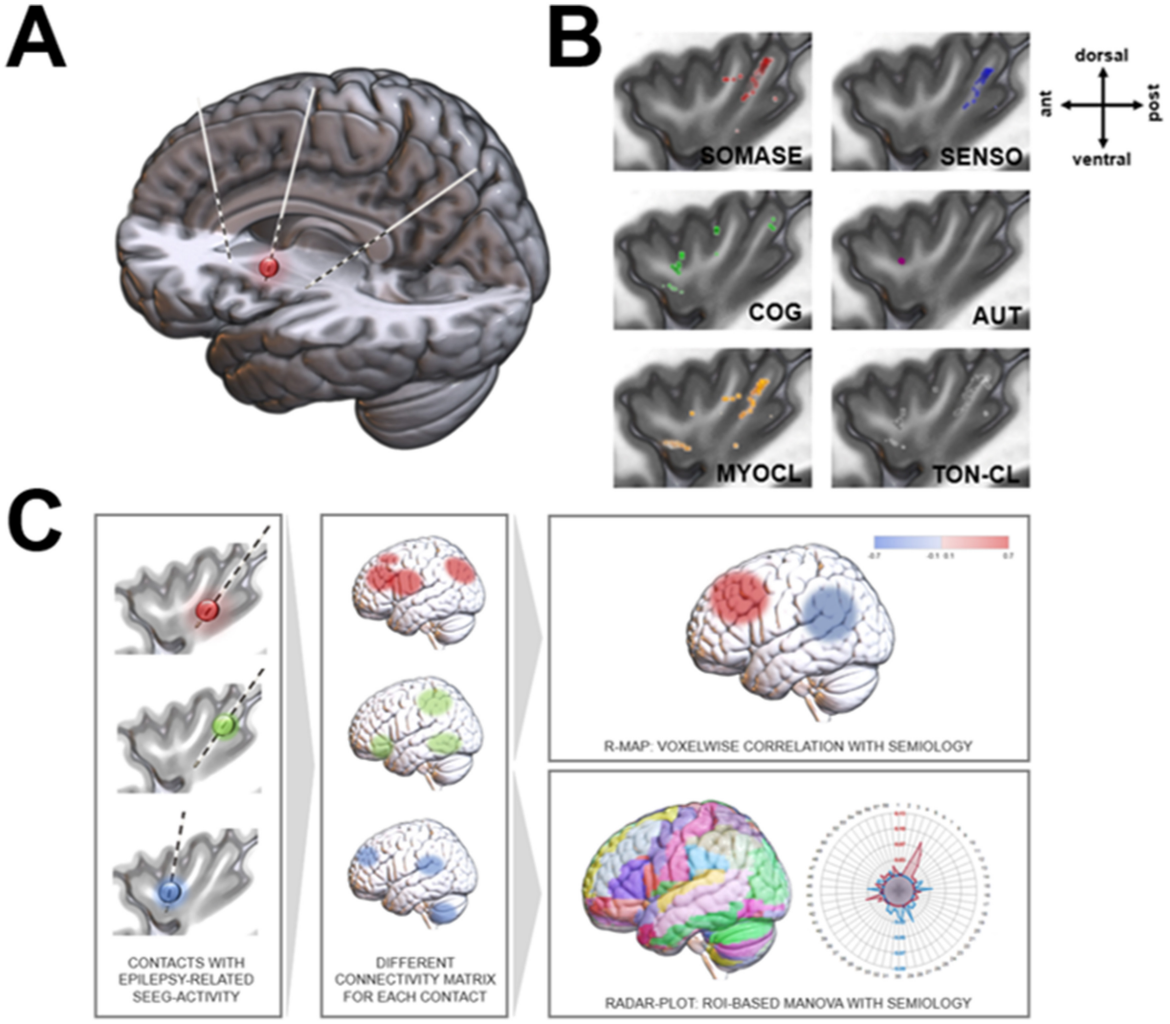
SEEG electrodes with insular epileptogenic onset activity. **(A)** Schematic trajectories in the anterior, middle and posterior insula via an anterior (AOA) or posterior oblique approach (POA). **(B)** Semiology-dependent structural distribution within the insula. **(C)** Schematic pipeline of data analysis. R-MAP, Regressor map; ROI, region of interest.

Electrode contacts with epileptiform discharges (SOZ-contacts as well as non-SOZ contacts) within the insula were detected and visualized with Matlab (MathWorks, Inc., Natick, MA, USA, R2018b), the SPM 12 (20) and Lead-DBS toolbox (https://www.lead-dbs.org) (21) as well as MRIcro/MRIcroGL (https://people.cas.sc.edu/rorden/mricro/mricro.html), retrospectively. The non-SOZ contacts of the insula were only included in the analysis if they showed epileptiform discharges in the early propagation phase. As soon as activity became visible on a large number of contacts of the implanted SEEG electrodes, these were not included. Pre- and postoperative MR scans were co-registered linearly using SPM 12(20) and then spatial normalized into the MNI_ICBM_2009b_NLIN_ASYM space (22). Potential brain shift in postoperative images was corrected by applying a refined affine transformation computed between pre- and postoperative images as implemented in the brain shift correction module in the Lead-DBS software (21). Afterwards, insular electrode contacts with epileptic activity were manually localized in postoperative MNI-normalized MR scans using MRIcro. Right-sided contacts were flipped to the left side, each active contact was separately saved as Nifti-file and insular activity patterns of seizure semiology were visualized (Figure 1). Localized contacts were automatically enlarged to a spherical region-of-interest (ROI_epi_) with a radius of 3.5 mm by a custom-written Matlab script and saved as Nifti-files for further connectivity analyses. The radius was chosen to reach the center of the adjacent contacts with the ROI edge (2 mm contact length and 1.5 mm intercontact distance) (23).

### Functional and structural connectivity analysis

The ROI_epi_ were imported into the Lead-DBS toolbox. Structural (DWI-based) and functional connectivity (fMRI-based) maps between each ROI_epi_ and voxels (isocentric 1 mm) in the rest of the brain were estimated using the Groupconnectome/Horn 2013 (structural) or PPMI 74_15 normative connectome datasets (functional, control group of the dataset) and the Lead Connectome Mapper software (Figure 1). The voxel-based analysis of the relationship between the structural and functional ROI_epi_ connectivity profiles and semiology (i.e., somatosensory, other sensory, autonomic, cognitive/emotional, myoclonic; 0: no, 1: yes) was performed with the *ea_Rmap.m* function (Lead-DBS toolbox). The *ea_Rmap.m* function is described in more detail below. This analysis provides for each voxel whether there is a positive or negative association between its connectivity to the ROI_epi_ and the semiology.

### Statistics

All analyses and statistical tests were performed using MATLAB (MathWorks, Inc., Natick, MA, USA), the Lead-DBS toolbox (https://www.lead-dbs.org) (21) and SPSS (IBM SPSS Statistics for Windows, Version 26.0. Armonk, NY: IBM Cor.). Data are referred to as the mean ± standard deviation (SD). *P* < 0.05 were considered significant.

**Figure 2:**
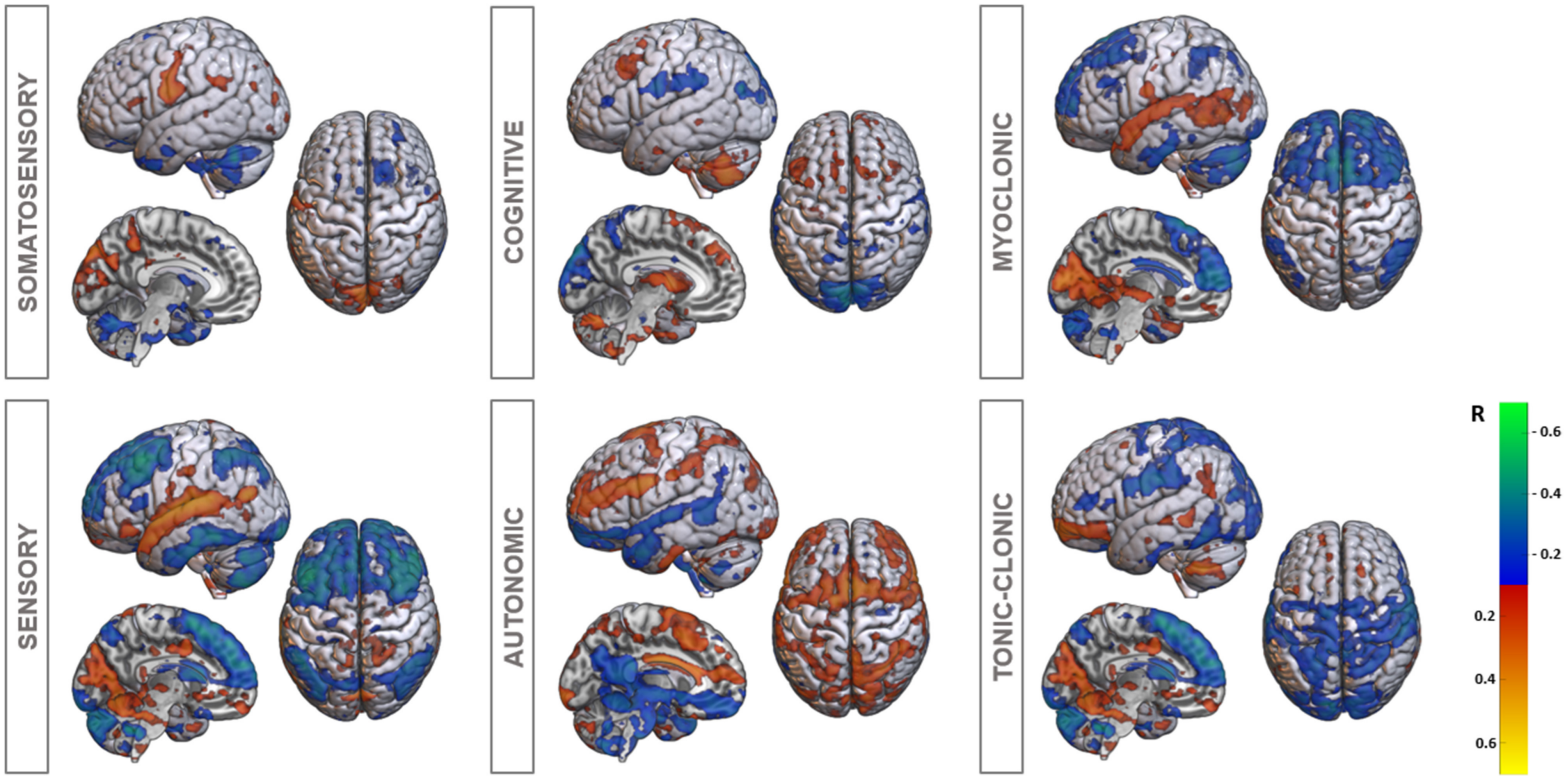
Voxel-based functional connectivity profiles (R-map) for different epileptogenic semiology. Hot colors (yellow to red; positive R-values) present voxels, whose connectivity is positively associated with the particular semiology. In cold color voxels (blue to green, negative R-values) a more negative connectivity is associated with the epileptogenic symptom.

**Figure 3:**
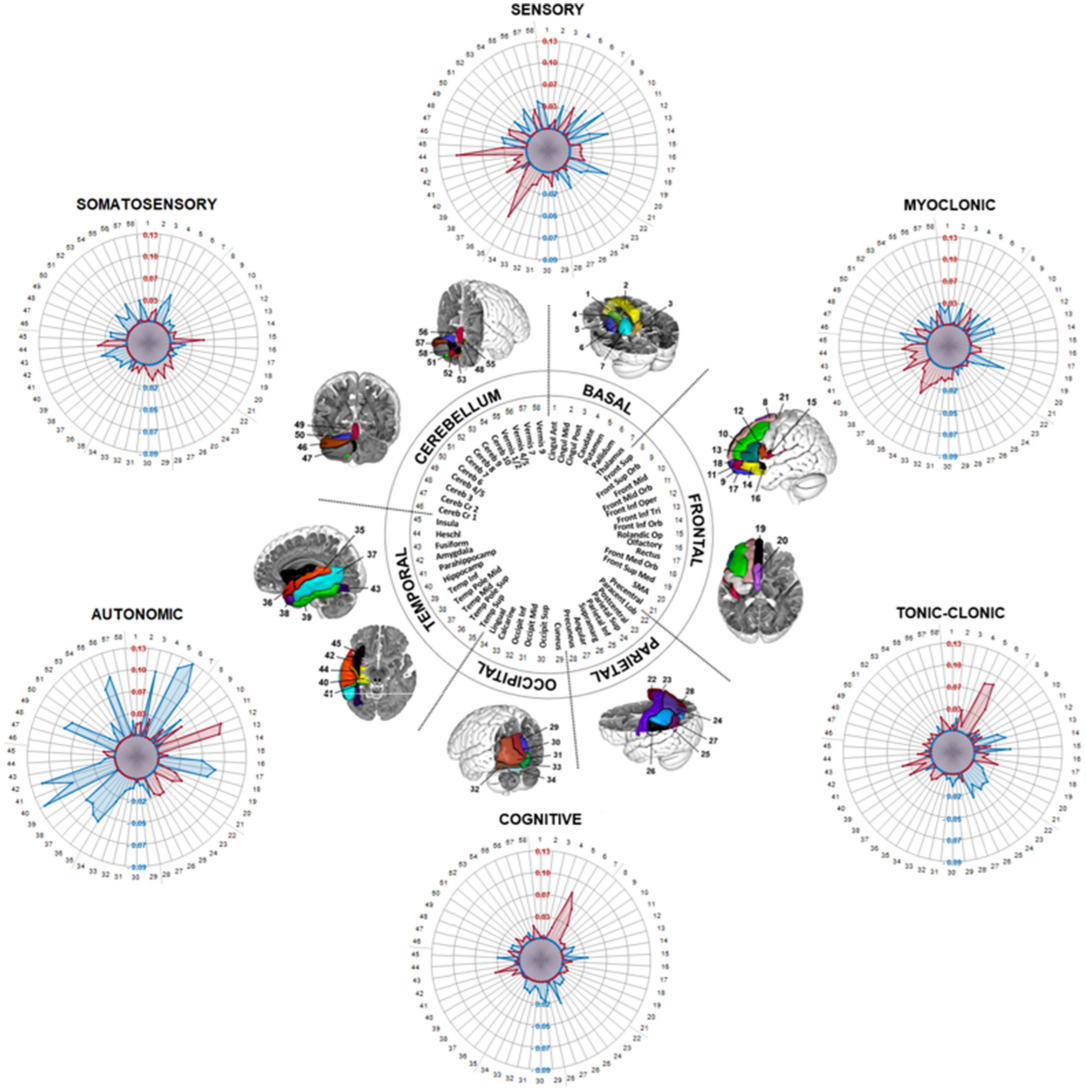
Radar plots showing different ROI-based functional connectivity clusters for epileptogenic semiology in the MANOVA. Semiology-dependent mean values of the overlap between 58 regions of the automated anatomical labeling atlas (AAL) and connectome maps of insular epileptiform contacts (ROI_epi_) are demonstrated (i.e., values in the radar plot demonstrate the difference between the overlap of semiology-positive ROI_epi_ with the AAL and the overlap of semiology-negative ROI_epi_ with the AAL). Positive values (red dots) denote a positive correlation and negative values (blue dots) imply negative connections.

Statistical analysis of the connectivity patterns in different seizure semiology were estimated by two different analyses: (a) Voxel-based analysis: Using the *ea_Rmap.m* function in Lead-DBS, ROI_epi_-specific connectivity maps were Spearman rank-correlated with the semiology (i.e., somatosensory, other sensory, autonomic, cognitive/emotional, myoclonic; 0: no, 1: yes) resulting in a voxel-based semiology-specific map (R-map) showing positive or negative associations with the semiology. R-maps of significant functional connections according to the Spearman correlation (*p* < 0.05) were visualized using MRIcroGL (Figure 2); (b) ROI-based analysis: ROI_epi_-specific connectivity maps were fused to the automated anatomical labeling atlas (AAL)(24) and after analyses of the overlapping volume of ROI-based maps and the 58 left hemispheric AAL regions (ROI_AAL_), the mean association of ROI_AAL_ to the semiology was calculated. Therefore, we applied multivariate analyses of variances (MANOVA) with SPSS (IBM SPSS Statistics for Windows, Version 26.0. Armonk, NY: IBM Corp.) to evaluate the effect of semiology patterns (i.e., SOMASE: somatosensory, SENSO: other sensory, COG: cognitive/emotional, AUT: autonomic, MYOCL: myoclonic, TON-CL: tonic-clonic) on connectivity matrices. ROI-based MANOVA was performed to control findings of the Voxel-based analysis. To ensure that results were not influenced by assumption violations, data were checked for outliers, homogeneity of variance–covariance matrices (Box's M test) and homogeneity of variances (Levene's test). MANOVA were followed by a univariate ANOVA to evaluate significance of semiology-associated differences in connectivity profiles (e.g., SOMASE 0=no vs. SOMASE 1 = yes). Connectivity group differences were visualized as radar charts using Matlab and MRIcro (Figure 3).

## Results

This retrospective analysis included a total of 118 insular contacts with epileptic discharges in 20 patients and 34 insular SEEG electrodes. 12/34 (35%) electrodes were located in the anterior insula (short anterior insular gyrus, apex and transverse insular gyrus), while 14/34 (41%) and 8/34 (24%) were implanted in the middle (short posterior insular gyrus) or posterior (long posterior insular gyrus) insula, respectively. In 8/20 patients the insula could be detected as SOZ by SEEG. After SEEG, 13/20 patients underwent resective epilepsy surgery (6/8 with insular SOZ, 7/12 with non-insular SOZ), while 2/20 received vagal nerve stimulation or electrocoagulation. In 3/20 patients a resective surgery was indicated but rejected by the patient due to the perioperative risks. Only in 2/20 resective surgery was not recommended due to multifocal SOZs. In 5/6 patients with insular SOZ and resective surgery, the seizure outcome—classified by the International League Against Epilepsy—was ILAE I (i.e., completely seizure free, no auras), only in 1/6 patient it was ILAE V (< 50% reduction of baseline seizure days to 100% increase of baseline seizure days; ± auras) (Table 1).

### Voxel-based structural connectivity patterns of the insula

We applied a structural (DWI-based) connectivity analysis to link different semiology types and insular SEEG contacts with epileptiform activity. This analysis reconstructed a common network junction in the posterior insula (PI) for sensory and somatosensory seizures (Figure 1). Motoric patterns (MYOCL and TON-CL) projected to the PI, and to a lower extent to anterior regions. In contrast, cognitive (COG) and autonomic (AUT) connections were found in the anterior insula (AI), with AUT seizures localized only in the dorsal AI (dAI), whereas COG foci were located in the dAI and ventral AI (vAI).

### Voxel-based functional connectivity analyses

Subsequently, we used a functional (fMRI-based) connectome to disentangle cortico-insular networks correlating with different semiology. Voxel-based analysis demonstrated significant patterns for all seizure types (R-map models: SOMASE: R = 0.39, *p* < 0.001; SENSO: R = 0.36, *p* < 0.001; COG: R = 0.44, *p* < 0.001; AUT: R = 0.31, *p* = 0.001; MYOCL: R = 0.33, *p* = 0.002; TON-CL: R = 0.34, *p* < 0.001). Functional connections are demonstrated in Figure 2. In summary, R-maps demonstrated positive correlations between insular SEEG contacts and rolandic opercular and postcentral regions in somatosensory epileptic symptoms (SOMASE), while there was a negative correlation to cerebellar regions. SENSO foci were positive correlated to the superior temporal gyrus and anticorrelated to the frontal and cerebellar cortex. A similar pattern was found in MYOCL seizures. However, correlations were not as strong as for the SENSO R-map. Moreover, there was no correlation to the postcentral gyrus in MYOCL. In contrast, TON-CL semiology was associated with negative connections to pre- and postcentral regions and positive correlated to the fronto-basal as well as medio-occipital cortex. Finally, COG symptoms were positive correlated to the cerebellum as well as basal regions and negative correlated to parieto-occipital atlas structures.

### ROI-based functional connectivity analyses

Separate MANOVAs were applied to 58 left-hemispheric regions of the AAL atlas in order to determine the group effect of distinct seizure types on the connectivity pattern. There was a significant multivariate main effect of group for all semiology types except MYOCL (Table 2).

**Table 2:** Multivariate analyses of variances (MANOVA) for ROI-based functional connectivity analyses.

	**Wilks' Λ**	**F_(58, 59)_**	**Partial η^2^**	*p*-value^*^
SOMASE	0.34	1.94	0.66	0.006
SENSO	0.28	2.56	0.72	< 0.001
COG	0.31	2.26	0.69	0.001
AUT	0.19	4.38	0.81	< 0.001
MYOCL	0.52	0.95	0.48	0.576
TON-CL	0.24	3.20	0.76	< 0.001

^*^p < 0.05 were considered significant.

Follow-up univariate ANOVAs revealed significant positive connections between the insula and temporal regions, especially the superior temporal gyrus [F_(1, 116)_ = 16.9, p < 0.001; partial η^2^ = 0.12] and the heschl gyrus [F_(1, 116)_ = 9.08, p = 0.003; partial η^2^ = 0.07] (Figure 3), in patients with sensory seizures (SENSO). Furthermore, negative connections to frontal brain areas were detected, e.g. the middle frontal gyrus [F_(1, 116)_ = 14.61, *p* < 0.001; partial η^2^ = 0.11], the medial superior frontal gyrus [F_(1, 116)_ = 18.06, p < 0.001; partial η^2^ = 0.14] and the triangular part of the inferior frontal gyrus [F_(1, 116)_ = 5.31, p = 0.023; partial η^2^ = 0.04]. In contrast, the pattern in somatosensory seizures (SOMASE) was characterized by significant positive connections of the insula to parieto-occipital regions like the postcentral gyrus [F_(1, 116)_ = 5.54, p = 0.02; partial η^2^ = 0.05], the Supramarginal gyrus [F_(1, 116)_ = 7.53, p = 0.007; partial η^2^ = 0.06] and the cuneus [F_(1, 116)_ = 10.8, p = 0.001; partial η^2^ = 0.09], while temporal and cerebellar regions were negatively connected.

The functional connectivity patterns in COG and TON-CL, however, were similar with positive associations between insula and basal regions, especially the putamen [F_(1, 116)_ = 12.67, p < 0.001; partial η^2^ = 0.1 and F_(1, 116)_ = 13.43, p < 0.001; partial η^2^ = 0.1] and pallidum [F_(1, 116)_ = 5.36, p = 0.022; partial η^2^ = 0.04 and F_(1, 116)_ = 12.96, p < 0.001; partial η^2^ = 0.1], and negative connections to parieto-occipital and supramarginal brain areas. Results showed significant connections to the amygdala [COG: F_(1, 116)_ = 5.85, *p* =0.017; partial η^2^ = 0.05; TON-CL: F_(1, 116)_ = 8.41 p = 0.004; partial η^2^ = 0.07]. Finally, analyses revealed very strong positive coactivation between the insula and the opercular part of inferior frontal gyrus [F_(1, 116)_ = 18.77, p < 0.001; partial η^2^ = 0.14] and the triangular part of the inferior frontal gyrus [F_(1, 116)_ = 14.42, p < 0.001; partial η^2^ = 0.11] in autonomic seizures (AUT), while we could demonstrate distinct negative connections to basal, cerebellar and temporal regions.

## Discussion

The aim of the present study was to investigate whether it is possible to decrypt insular epilepsy networks by combining insular SEEG data with connectomics. This idea is based on an altered understanding of epilepsy as a network- rather than lesion-based disease (1, 4, 7). Furthermore, it ties in with connectome-based analyses in movement disorders and deep brain stimulation (DBS), which have provided significant insights into stimulation effects and disease processes (25). However, our study extends these approaches by a transparent ROI-based MANOVA in addition to the voxel-based analysis. We were able to demonstrate the feasibility of such an analysis to describe the association between cortico-insular networks and seizure semiology. Epileptic foci were found in the posterior insula for sensory, somatosensory and motor seizures, while cognitive and autonomic symptoms were related to the anterior insula. We identified insular connections especially to the superior temporal gyrus and the heschl gyrus in sensory seizures and projections to the somatosensory cortex in somatosensory seizure onsets. Insula-basal ganglia pathways were found in cognitive seizure manifestations, while insular connectivity to fronto-basal regions were strongest in autonomic seizures. In conclusion, our findings demonstrate that the seizure semiology is mirrored in the functional connectivity of insular epileptic activity.

### Insular connectivity clusters

Our structural connectivity analysis is consistent with the known functional and cytoarchitectonic parcellation of the insular cortex along the rostrocaudal anatomical axis with sensorimotor areas localized in the posterior and cognitive/autonomic semiology mainly in the anterior insula (10). While AUT regions could be detected only in the dAI between the anterior short gyri (ASG) and middle short gyri (MSG), COG was located in both, vAI and dAI. In previous studies, higher-level cognitive processes (e.g., speech and attention) were predominantly observed in the dAI, while emotional functions were located in the vAI (26, 27).

Subsequent examination of functional connectivity patterns revealed that AUT regions of the insula are positively connected/correlated to/with fronto-basal regions. These results correspond to previous studies in healthy subjects and epilepsy patients demonstrating connections between the dAI and frontal operculum (26, 28, 29). SEEG contacts associated with COG seizures, which were also located in ASG and MSG, revealed interactions to the amygdala, as well as the putamen, pallidum and thalamus. While Almashhaiki et al. (28) could not detect an insular association to the amygdala, numerous studies have verified such connections, especially as part of the salience network (10, 27, 29–31). In concordance, COG seizures were anti-correlated to insular-supramarginal connections. The supramarginal gyrus, however, is a major node of the default mode network, which typically is anti-correlated to the salience network (27). Furthermore, our results are consistent with connectivity analysis in genetic generalized epilepsy (GGE) which demonstrated, that GGE patients with interictal epileptogenic discharges exhibited an increase in functional connectivity in the bilateral caudate nucleus, putamen, and insula compared to GGE patients without interictal discharge (32). In contrast, we could not determine significant networks between ASG/MSG and the hippocampus, which were described in comparable studies (28, 33).

The connectivity cluster of sensorimotor seizure areas, located in the posterior insula, fits well with previous results, which detected an association of the posterior long gyrus (PLG) to the perisylvian region with a higher connectivity rate to the temporal and parietal operculum, than to the frontal operculum (28). In particular, the connection to the superior temporal gyrus was seen in sensory seizures encountered in the PLG and was also observed in other structural and functional connectivity studies (31, 34). Moreover, the connectivity to primary somatosensitive regions observed in sensory seizures is also pre-recognized (31).

### Network semiology

Considering epilepsy as network disease implies that seizures of different brain regions with comparable semiology affect the same pathways. This theory is supported by a study of Bonini et al. (35) that investigated network activity in frontal lobe seizures with different symptoms. Frontal seizures with negative emotional expressions exhibited increased activity in the amygdala, as was demonstrated for insular epileptic activity in our study. Furthermore, several studies in temporal lobe epilepsy revealed a high interconnectivity of the ipsilateral hippocampus (a typical seizure onset region in TLE) as well as of the superior temporal lobe to the insula (36). This might explain common sensoric phenomena (e.g., olfactory, gustatory) in both epilepsy forms. However, results of the mentioned studies are only partially consistent. Whereas tonic-clonic seizures were mainly associated with negative connections to pre- and postcentral regions and positively correlated to the fronto-basal as well as medio-occipital cortex in our analyses, Bonini et al. (35) demonstrated increased positive connections to the primary motor cortex in generalized tonic-clonic seizures. These inconsistency may be related to an imprecise specification of seizure semiology types in our study because of the small cohort. Studies with larger numbers of patients and different SOZs with the same semiology are necessary to better characterize network semiology in future.

### Future perspectives

Summarizing our results, SEEG-based connectome analysis may not only contribute to encrypt symptom-specific networks but also to define targets for deep brain stimulation or lesion therapy and to predict their effect in pharmaco-resistant epilepsy. So far, only DBS of the anterior nucleus of the thalamus has been approved for refractory epilepsy (37). Connectome-based outcome analysis demonstrated different lead locations and connectivity profiles for patients with a good stimulation outcome in contrast to patients with a poor outcome (38). However, connectome-based network analysis may help to identify critical nodes within dynamic epileptic networks for DBS (39). Notably, the present study has identified insular connections to the basal ganglia. Studies in animals have already demonstrated a positive effect of ventral pallidum stimulation on seizure frequency and expression (40). Besides, previous studies described the amygdala for emotion regulation (41) and the hippocampus for seizure reduction in epilepsy (42) as promising DBS targets (39). However, further studies are necessary to clarify the effectiveness or requirements (e.g., seizure type, SOZ) for such stimulation. Further scientific progress in this context may be achieved by extending the presented analysis technique as graph-theory based brain network hub analysis and in patient-individual fMRI (43).

### Limitations

The present study represents a retrospective SEEG-based connectome analysis. Thus, main limitations are due to connectomics, i.e., (i) normative connectomes do not consider individual differences in brain connectivity. Previous studies with patient-specific connectivity data have shown that patients with epilepsy may have abnormal nodes, a reduction in fiber density, and altered network structures compared to healthy patients (44). Thus, connectivity patterns of normative connectomes might not be valid for the connectomes of epilepsy patients. However, individualized connectomes may also be associated with significant limitations in signal-to- noise ratio and reproducibility. Therefore, normative connectomes may have an advantage in this context; (ii) MRI-based connectivity techniques (e.g., fMRI) are not sensitive to the directionality of connectivity (e.g., inputs vs. outputs)(45); (iii) The choice of parcellation atlas has an effect on topological results (46). Finally, our results are limited by the fact that analysis was not performed separately for the left and right hemisphere or only SOZ contacts (but also non-SOZ within the insula) due to the cohort size. However, previous studies suggest that there may be side differences in insular connectivity (31). Furthermore, right- and left-lateralized epilepsy may have distinct functional connectivity patterns and structural and functional connectomes might be asymmetric (47). Therefore, future studies with larger patient cohorts should consider this aspect. In addition, the consideration of interictal spike propagation and the use of feature extraction methods and machine learning algorithms in the analysis would be conceivable (48, 49).

### Conclusion

Different semiology of insular epileptogenic activity are mirrored in the functional connectivity network of the insular epileptic discharges. Combining SEEG and connectomic analyses, therefore, could provide additional information about seizure propagation within the epilepsy network and might enable new treatment options in future.

## Data Availability

The data analyzed in this study is subject to the following licenses/restrictions: data can be provided on reasonable request. Requests to access these datasets should be directed to kathrin.machetanz@med.uni-tuebingen.de.
