# Supplementary material for: Connectome-based disentangling of epilepsy networks from insular stereoelectroencephalographic leads
Source: Front Neurol. 2025 Jan 3;15:1460453. doi: 10.3389/fneur.2024.1460453 (PMC11738935; doi:10.3389/fneur.2024.1460453)
Supplement: Supplementary file 1 [file Table_1.docx]

**Supplementary Table: Patients’ seizure characteristics**

| **Pat** | **contacts** | | | **SOMASE** | **SENSO** | **AUT** | **COG** | **MYOCL** | **TON-CL** |
| --- | --- | --- | --- | --- | --- | --- | --- | --- | --- |
|  | ANT | MID | POST |  |  |  |  |  |  |
| **1** | 4 |  |  |  |  |  |  |  |  |
| **2** | 3 | 4 |  |  |  |  |  |  |  |
| **3** | 2 | 2 |  |  |  |  |  |  |  |
| **4** |  | 1 |  |  |  |  |  |  |  |
| **5** | 4 |  |  |  |  |  |  |  |  |
| **6** |  | 4 |  |  |  |  |  |  |  |
| **7** |  | 4 | 6 |  |  |  |  |  |  |
| **8** | 2 | 2 |  |  |  |  |  |  |  |
| **9** | 2 | 4 | 2 |  |  |  |  |  |  |
| **10** | 2 |  |  |  |  |  |  |  |  |
| **11** |  |  | 1 |  |  |  |  |  |  |
| **12** | 1 |  |  |  |  |  |  |  |  |
| **13** |  |  | 2 |  |  |  |  |  |  |
| **14** |  | 1 |  |  |  |  |  |  |  |
| **15** | 7 | 3 | 3 |  |  |  |  |  |  |
| **16** |  | 4 |  |  |  |  |  |  |  |
| **17** | 3 |  |  |  |  |  |  |  |  |
| **18** |  | 7 | 6 |  |  |  |  |  |  |
| **19** |  | 2 |  |  |  |  |  |  |  |
| **20** | 10 | 10 | 10 |  |  |  |  |  |  |

Green marked cases demonstrate cases with insular seizure onset zone (SOZ, n=8). Red squares demonstrate that the patient has a specific seizure type, while gray squares show that this seizure type was not present. ANT, MID and POST columns describe the number of contacts in the anterior, medial and posterior insula. SOMASE: somatosensory; SENSO: sensory; AUT: autonomic; COG: cognitive; MYOCL: myoclonic; TON-CL: tonic-clonic
